# Whole organ vascular casting and microCT examination of the human placental vascular tree reveals novel alterations associated with pregnancy disease

**DOI:** 10.1038/s41598-017-04379-0

**Published:** 2017-06-23

**Authors:** Toluwalope O. Junaid, Robert S. Bradley, Rohan M. Lewis, John D. Aplin, Edward D. Johnstone

**Affiliations:** 1 Maternal and Fetal Health Research Centre, Faculty of Biology, Medicine and Health, University of Manchester, Manchester, United Kingdom; 2 Henry Moseley X-ray Imaging Facility, School of Materials, University of Manchester, Manchester, United Kingdom; 3 Faculty of Medicine, University of Southampton, Southampton, United Kingdom

## Abstract

Experimental methods that allow examination of the intact vascular network of large organs, such as the human placenta are limited, preventing adequate comparison of normal and abnormal vascular development in pregnancy disease. Our aims were (i) to devise an effective technique for three-dimensional analyses of human placental vessels; (ii) demonstrate the utility of the technique in the comparison of placental vessel networks in normal and fetal growth restriction (FGR) complicated pregnancies. Radiopaque plastic vessel networks of normal and FGR placentas (n = 12/group) were created by filling the vessels with resin and corroding the surrounding tissues. Subsequently, each model was scanned in a microCT scanner, reconstructed into three-dimensional virtual objects and analysed in visualisation programmes. MicroCT imaging of the models defined vessel anatomy to our analyses threshold of 100 µm diameter. Median vessel length density was significantly shorter in arterial but longer in venous FGR networks compared to normals. No significant differences were demonstrable in arterial or venous tortuosity, diameter or branch density. This study demonstrates the potential effectiveness of microCT for *ex-vivo* examination of human placental vessel morphology. Our findings show significant discrepancies in vessel length density in FGR placentas. The effects on fetoplacental blood flow, and hence nutrient transfer to the fetus, are unknown.

## Introduction

Successful development of the fetoplacental vasculature is essential to sustain healthy pregnancy. In a well-developed human placenta, the umbilical vessels – two arteries and one vein – travel together within the umbilical cord which inserts near the centre of the placenta^1^. From the cord insertion, each vessel branches extensively across the chorionic plate, fusing with locally formed vessels within the villi^2, 3^, down to the terminal villi where feto-maternal exchange primarily occurs^4^. The branches of each vessel form a distinct network across the chorionic plate and within the villi. Structural deficits in the vessel networks may result in deficient nutrient transfer and ultimately complications such as fetal growth restriction (FGR).

FGR is characterised by failure of a fetus to attain its genetically endowed growth potential. It is of global significance, complicating more than 8% of pregnancies^5^. The placenta plays a central role in the pathogenesis of FGR^4^ and although it can tolerate localised structural and functional impairments^6^, whole organ function is the critical determinant of nutrient and oxygen supply to the fetus. Extraction of holistic information on the placental vascular network is therefore necessary to identify disease-associated changes that are likely to be of functional significance. Moreover, as there are variations in vascularity of samples from different regions of the placenta^7^, evaluation of the whole organ would better represent its entire vascularity. To date, whole organ examination and quantification of the placental vascular tree remains an experimental challenge. Corrosion casting of placental vessels permits three dimensional (3D) examination and quantification of the vessels^7, 8^, but the small calibre vessels of the villi are difficult to quantify accurately from casts by direct measurement. Also, while the villi vessels are accessible by histology, 3D reconstruction analyses of histological sections limit obtainable data to small tissue samples hence do not represent the vascularity of the whole organ. Moreover, histology has been shown to be unreliable for interpretation of the three dimensional (3D) structure of placental trees^9^.

In this study we aimed to address this problem using a combination of corrosion casting and microcomputed tomography (micro-CT) imaging to achieve a fuller account of placental vascular morphology. Micro-CT imaging permits 3D identification and quantification of anatomical trees. In previous studies, it has provided a detailed view of the vasculature in the kidney^10, 11^, heart^12^, brain^13^ and placenta^14^ of animals. To our knowledge the technique has yet to be used in the study of the human placental vascular tree in its entirety. Having performed micro-CT on vessel casts derived from human placentas we then aimed to compare placental arterial and venous vessel networks in placentas from uncomplicated pregnancies and pregnancies complicated by fetal growth restriction (FGR).

## Materials and Methods

### Ethics statement and sample preparation

All placental tissues were obtained from women who delivered at St. Mary’s Hospital, Manchester, United Kingdom, in accordance with approved guidelines at the hospital. Ethical approval was granted by the Local Research Ethics Committee [Greater Manchester Central (REC 08/H1010/55)] which is part of the UK National Research Ethics Service. Informed written consent was obtained from all placenta donors prior to delivery and normal and FGR placentas were obtained within 30 minutes of delivery. FGR was defined as individualized birth ratio (IBR) ≤5^th^ centile (Table 1). Before imaging, all samples were processed in the Maternal and Fetal Health Research Centre Laboratory, Manchester, with appropriate licence for handling human tissue. Imaging and Avizo analyses were done in the Henry Moseley X-ray Imaging Facility, Manchester. Analyze analyses was done in the Wolfson Molecular Imaging Centre, Manchester.

**Table 1:**
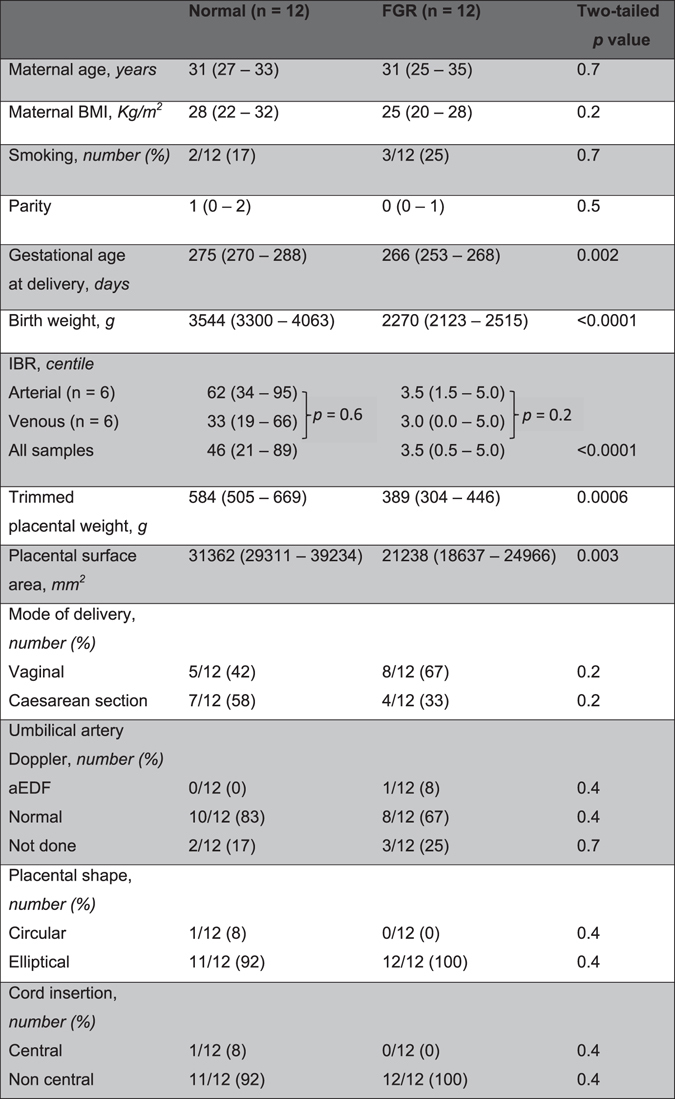
Demographic, clinical and gross placental examination details of study participants

Data shown are median and interquartile range (IQR) in parenthesis or in a number with percentage in paranthesis as appropriate. *p* < 0.05 is significant; Mann Whitney U test. BMI, IBR and aEDF represent body mass index, individualised birth weight ratio and absent end diastolic flow respectively. ‘Arterial’ and ‘venous’ refer to the vascular compartments casted for the respective placentas.

### Pre imaging processing: Corrosion casting

The fetal membranes were trimmed off each placenta before weighing the placental disc. The umbilical cord was not trimmed off because it was required for vessel cannulation prior to corrosion casting. The fetal and maternal sides of each placental disc were photographed. Placental surface area (PA) was determined by uploading a photograph of each placental disc on Image Pro Plus software (Media Cybernetics Inc, USA) and tracing out an outline of the disc. The software computed the surface area for each outline.

The fetoplacental vasculature was perfused with a radiopaque methylmethacrylate-based casting material [Batson’s No. 17, Anatomical Corrosion Kit (Polysciences Inc, Germany)] as described in published corrosion casting protocols^7, 15, 16^. The cord was clamped immediately after delivery to ensure the vessels were kept dilated. For preparation of venous casts, the umbilical vein was cannulated within the cord, while for arterial casts, one of the umbilical arteries was cannulated about 5 cm before cord insertion (ahead of the Hyrtl’s anastomosis^17^) using a 20 G cannula held in place with a suture. The two umbilical arteries are connected by the Hyrtl’s anastomosis near the cord insertion in most human placentas^17, 18^. Therefore, cannulating one of the arteries ahead of the anastomosis permitted flow of perfusate into both arteries. About 20 ml of 5000iu/L heparin in pre-warmed phosphate buffered saline (PBS) was infused to prevent intravascular coagulation. The PBS had been warmed to promote flow-mediated vasodilation. After 10 minutes, freshly prepared casting material (at room temperature) was manually injected through the cannula until back pressure prevented further injection. The cord was then clamped below the point of cannulation, to prevent leakage of the polymer. The placenta was left overnight on a polythene sheet on ice to lower the temperature, allowing polymerisation to occur at a more uniform rate. The following day, the whole cast placenta was immersed in 500 ml of 20% w/v potassium hydroxide (KOH; Fisher Scientific, Lutterworth, UK) within a gasket-sealed tub in a water bath at 40 °C. KOH solution was changed 6 hourly until the tissue was completely corroded. The solution was then replaced with distilled water for 6 hours to rinse off the KOH. The rinsed cast was air dried and photographed. The success rate of the casting experiment was approximately 81%, 64%, 60% and 64% for the normal arterial, normal venous, FGR arterial and FGR venous sample groups respectively. A total of 12 normal (6 arterial, 6 venous) and 12 FGR (6 arterial, 6 venous) vascular casts were included in the study.

### Image acquisition and processing

3D datasets were acquired for each cast using a high resolution micro-CT machine (Nikon metris XTH225, Nikon Metrology NV) fitted with a 225/320 keV x-ray CT source, a rotating stage and a 2000 × 2000 Perkin Elmer detector, and controlled by Nikon’s Inspect-X software. The x-ray source was a microfocus tube emitting x-rays in cone beam geometry. The cast, placed on the stage, was rotated over 360° at angular increments of 0.2° around the vertical axis with the whole cast within the field of view as scanning was performed with the x-ray source voltage set to 42 kV. Maximum magnification and exposure time varied depending on each specimen’s circumference, which had to be within the cone beam of irradiation. About 2000 slice views were generated for each cast. CT data acquired by Inspect-X were received via an application (CT Agent, Metris, UK) which wrote the files to disk, synchronising them with reconstruction software (CT Pro 2.0, Metris, UK). Reconstructed voxel sizes were in the range 0.08 to 0.1 mm depending on the size of the cast. Vessels below 100 µm in diameter were excluded from the analysis to be consistent across samples. The contrast to noise ratio of the CT data was sufficient to enable vessels down to 100 µm diameter to be segmented automatically. Following 3D reconstruction of the CT projections into a single volume 3D virtual object on CT Pro, a MATLAB (MathWorks, Massachusetts, USA) algorithm was used to export the data into *.hx* and *.tiff* format compatible with volume rendering in Avizo (Avizo 8.0, FEI Visualization Sciences Group, Konrad-Zuse-Zentrum fur Informationstechnik Berlin (ZIB) and FEI, SAS) and Analyze (Analyze 12.0, AnalyzeDirect Inc, Biomedical Imaging Resource (BIR), Mayo Clinic, USA) 3D analysis software packages respectively. Images were examined for evidence of artefacts or breakages which were not found above 100 µm.

### Image analysis

#### Three-dimensional vascular tree analyses in Avizo software

Each 3D dataset was volume-rendered in Avizo. Background noise was removed from the image using the ‘orthoslice’ tool and colourmap adjustments. Analysis was carried out in 800 × 500 × 300 voxel subvolumes (depending on the size of each cast, 6–9 subvolumes were extracted from each image). The vessels in each subvolume were segmented using the ‘image segmentation’ tool. Segmented vessels were skeletonised, rendering them in different colours based on their diameter. Nodes placed along the path of each vessel, representing a site of branching and/or any deviation of the vessel from a straight path, separated the vessels into various segments from which measurements of morphological parameters such as vessel length, diameter and tortuosity/loopiness were derived. Data obtained from all subvolumes in each cast were added together.

#### Three-dimensional vascular tree analyses in Analyze software

For each cast, all micro-CT image slices were imported as a single volume object into the Analyze workspace. Once loaded, the object was rendered for thresholding to generate a tree-like structure, which represents the skeleton of all the vessels in the original cast. The software placed blue nodes at branch points and red nodes at terminal points of branches in the tree. These points represent the farthest extent of penetration of methacrylate into the respective vasculature. From the generated trees, the total number of branches, number of true branches, number of branch levels and number of branches per branch level in each cast was extracted.

### Statistical analyses

Statistical analyses were conducted using GraphPad Prism® 7 (GraphPad Software, Inc., USA). Unless otherwise stated, data were represented as box and whiskers showing the median, range and interquartile range, compared by Mann Whitney test and a p value of <0.05 was considered to be statistically significant. In all, twenty-four samples were analysed [12 normal (6 arterial, 6 venous) and 12 FGR (6 arterial, 6 venous)]. Previous analyses showed that differences in the human placental macrovasculature and microvasculature are detectable at similar sample size^7^.

## Results

### Measured vessel biometry outputs

**Figure 1:**
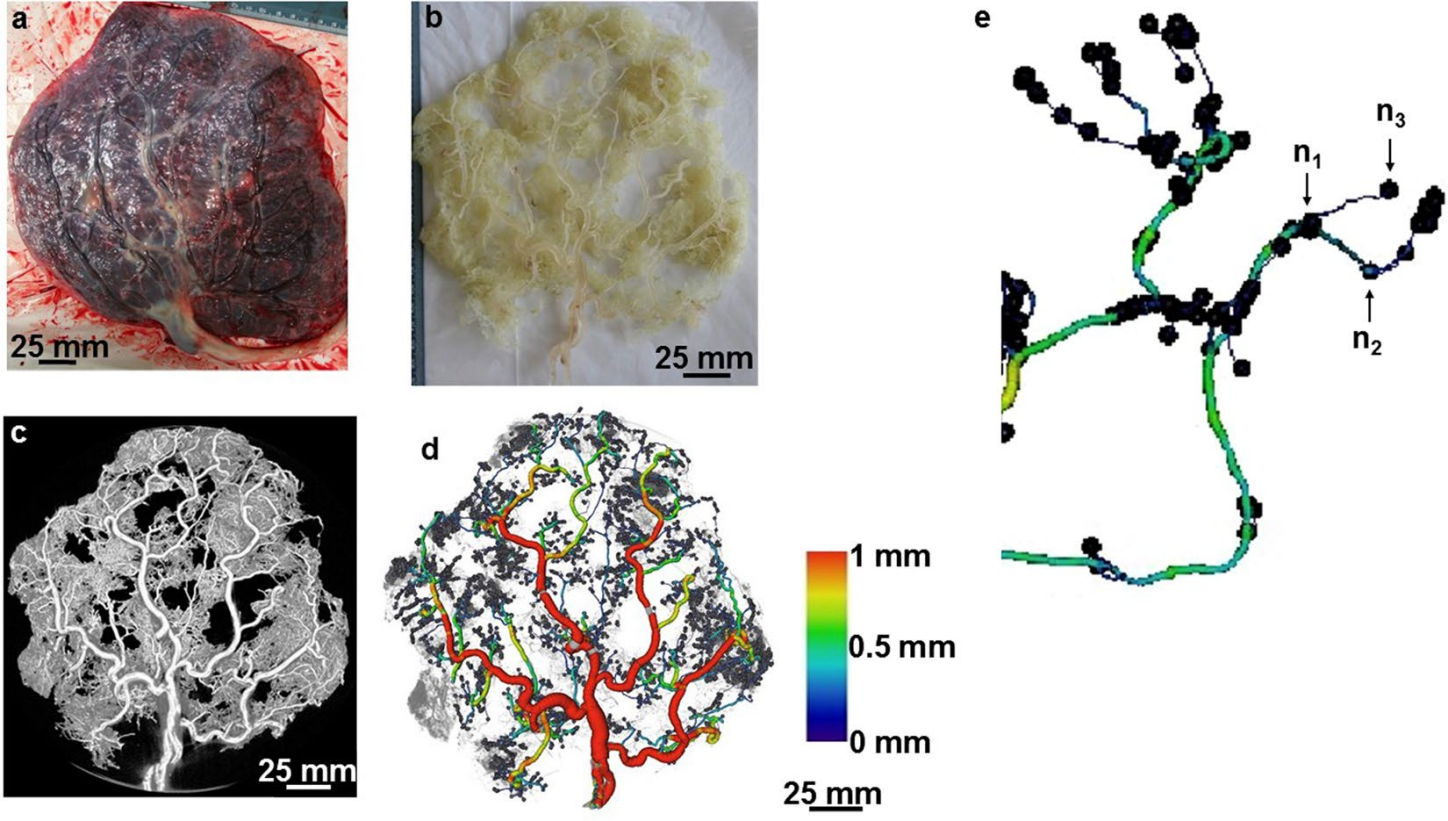
An example of a scanned and analysed arterial cast of a normal placenta. The images show an arterial cast (**b**) following corrosion casting of a normal placenta (**a**), a 3D reconstructed image of the arterial cast following micro-CT scanning (**c**) and analysis in Avizo (**d**). Vessels are coloured based on their diameter. The image in panel e is a section of an analysed cast zoomed to show nodes placed at a true vessel branch point (n_1_), a point where vessel course deviated from a straight path (n_2_) and a terminal point (n_3_) along the vasculature. Vessel diameter declined as the vascular tree branching progressed. Scale 25 mm.

**Figure 2:**
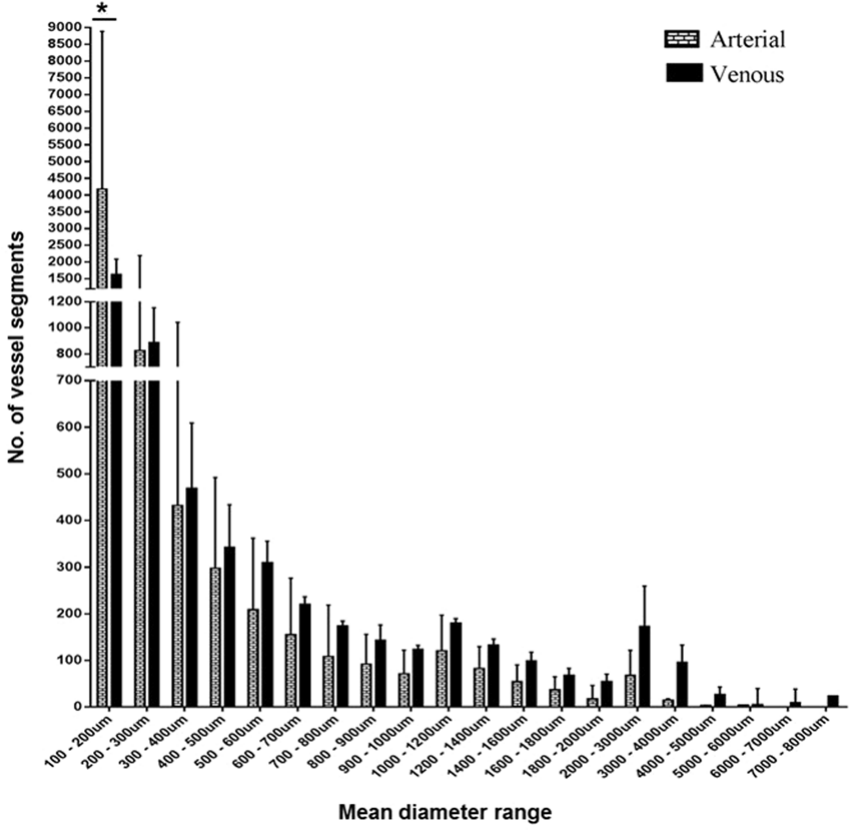
The number of vessel segments in different diameter ranges in the normal arterial and venous casts. There were more vessels (arterial and venous) in the smallest diameter group. The number of vessel segments declined progressively from the smallest to the largest diameter ranges. There were significantly fewer segments within the 100–200 µm diameter range in the venous compared to the arterial casts. Median + Interquartile range of n = 12 (6 arterial, 6 venous) casts shown. Two-way RM Anova with Sidak’s multiple comparison test. Alpha value = 0.05, *marks two-tailed *p* value = 0.0001.

**Figure 3:**
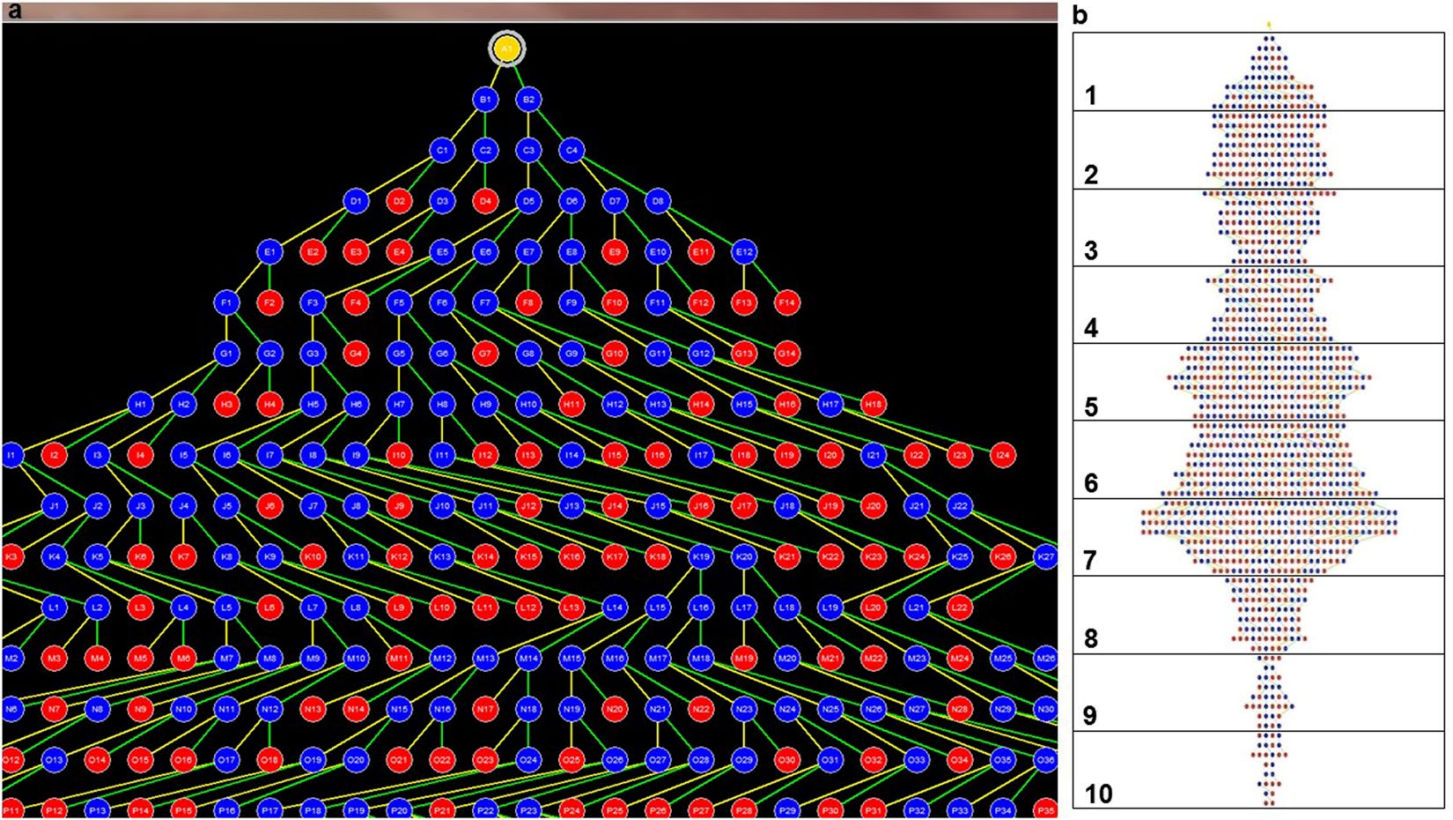
Vessel tree maps generated on Analyze. Panel (a) is a screenshot showing part of a tree map of vessels. The yellow dot at the top represents the root of all the vessels (umbilical cord insertion point). True branch points and terminal branch points are marked in blue and red dots respectively. Each point is labelled with alphabets designating the branch generation/level and numbers designating the branch number at each level. Panel (b) shows a sample tree map grouped into decile (ten groups) of branching. In this example, the total level of branching (excluding the root) was eighty. This was divided into ten groups containing eight levels each. The number of branches in each group was counted.

Reconstruction of scan slices acquired by micro-CT of vessel casts permitted 3D visualisation of most of the placental vascular tree and production of a digital replica for analysis (Fig. 1). We observed the casts on a stereomicroscope and the diameter of the smallest terminal vessels in the casts was ~10 µm. However, minimum vessel resolution from CT images was ~80 µm (diameter) from a whole placental cast measuring ~17 cm (widest diameter). Because there were breakage points in some casts especially at the level of the small, fragile branches, only vessels ≥100 µm in diameter were included in the analyses. Following segmentation and skeletonisation of reconstructed images using Avizo software (Fig. 1d), the number of vessel segments, length and diameter of each segment, and the total length of all vessel segments were generated. In the normal placental casts there was an expected progressive decline in number of vessels from smaller to larger arteries and veins to the umbilical cord insertion; there were significantly fewer venules (100–200 µm) compared to arterioles of similar diameter (Fig. 2). This difference persisted when controlling for variation in placental weight and surface area (data not shown). In Avizo, nodes were placed at true vessel branch points, points where vessel course deviated from a straight path and at terminal points (Fig. 1d,e). Total node number therefore represents the sum of branch points and vessel bends in the feto-placental vasculature. A further analysis package, Analyze, was used to determine branch point alone. Skeleton tree maps demonstrating sequential branching levels for each microCT dataset were therefore generated (example in Fig. 3). Analyze allowed determination of the tortuosity of the vessels within the fetal arterial and venous trees. Tortuosity was defined as total node number (Avizo) minus true branch number (Analyze).

### Demographic and gross placental features in normal and FGR placentas studied

Having developed analysis tools to examine digital casts of placental fetal vasculature at a whole organ level, we compared vessel morphology in a selection of placentas from normal and FGR pregnancies. The demographic and clinical details of the women and newborns whose placentas were used are shown in Table 1. At delivery, the median gestational age, birth weight, individualized birth ratio (IBR), placental weight and placental surface area were significantly different between the normal and FGR-complicated clinical groups. Maternal age, body mass index, parity, smoking status, mode of delivery and early gestation Doppler findings were not different. There were no differences in the birth centile of the placentas used for arterial or venous casts for either the normal or FGR populations. Placentas from the normal and FGR clinical groups differed significantly. Although grossly, placental shape and cord insertion were not different, median surface area in normal placentas was 31362 mm^2^ (interquartile range [IQR] 29311–39234 mm^2^) compared to 21238 mm^2^ (IQR 18637–24966 mm^2^) in FGR; p = 0.003. Similarly, median placental weight was 584.00 g (IQR 504.60–668.50 g) and 389.00 g (IQR 304.30–446.00 g); p = 0.0006 for the normal and FGR groups respectively.

### Measurements of vascular morphology

**Figure 4:**
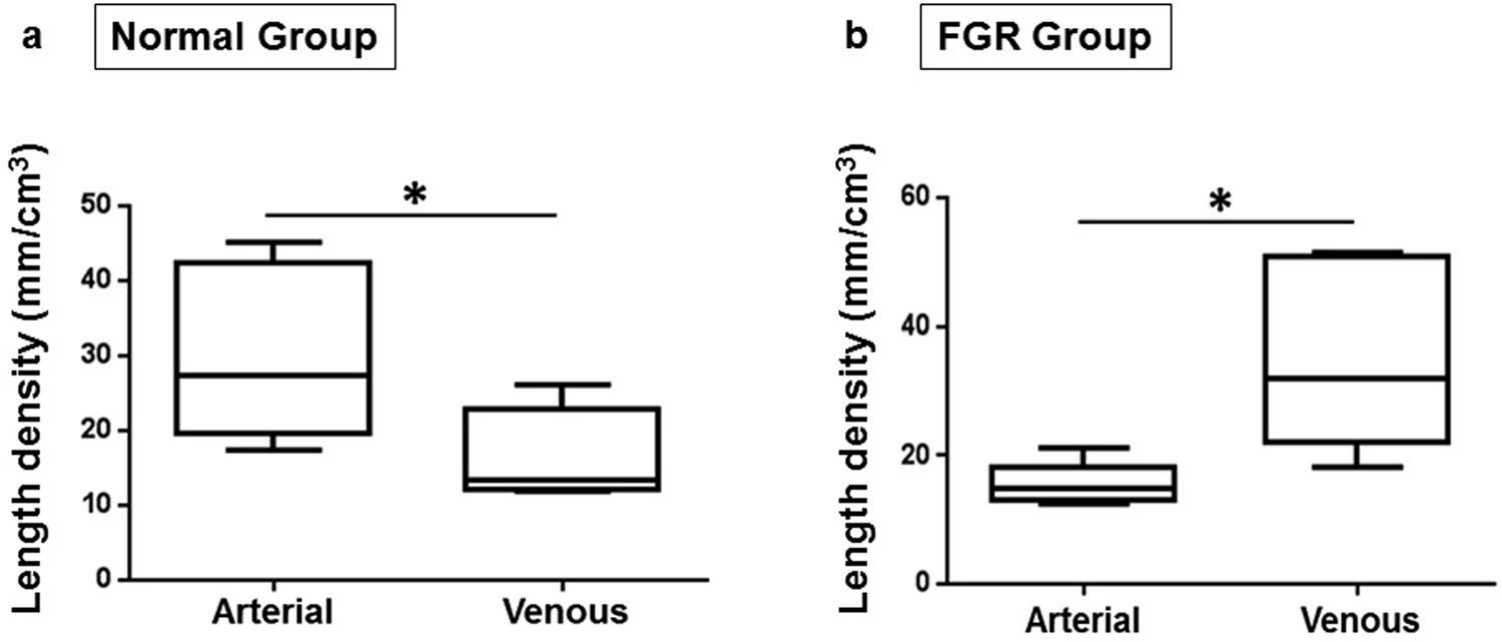
Arterial and venous length density in normal and FGR casts. Panels (a) and (b) represent the vessel length density in the normal and FGR casts respectively. In the normal casts, arterial paths are longer than venous paths. The opposite was observed in the FGR casts; these had longer venous paths. Data presented as boxplots showing the median, range and interquartile range. n = 12 normal (6 arterial, 6 venous) and 12 FGR (6 arterial, 6 venous) casts. Mann Whitney test, *marks two-tailed *p* value = 0.04 and 0.004 in a and b respectively.

**Figure 5:**
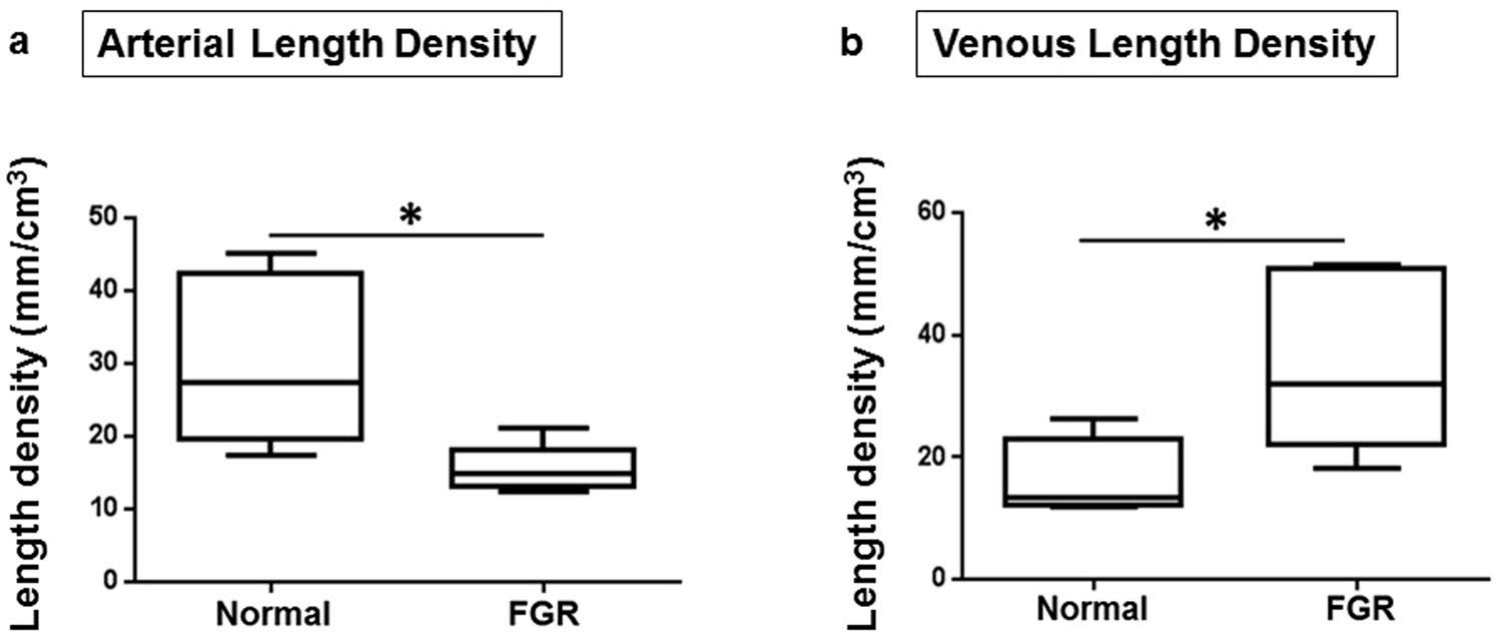
Arteries are shorter and veins are longer than normal in FGR casts. Panels a and b represent (a) the arterial length density and (b) venous length density respectively. Compared to normal casts, arterial path was significantly shorter in FGR (**a**). However, the converse is true for the venous network (**b**). Data presented as boxplots showing the median, range and interquartile range. n = 12 normal (6 arterial, 6 venous) and 12 FGR (6 arterial, 6 venous) casts. Mann Whitney test, *marks two-tailed *p* vaule = 0.009 and 0.03 in a and b respectively.

Vascular parameters measured for each placenta were expressed per cm^3^, assuming 1 g = 1 cm^3^, as reported in previous 3D morphometric studies^19, 20^. In normal placentas, the median arterial length density was 27.29 mm/cm^3^ (IQR 19.65–42.44 mm/cm^3^), significantly greater than the median venous length density of 13.39 mm/cm^3^ (IQR 12.18–22.94 mm/cm^3^); p = 0.04 (Fig. 4a). The converse was noted in FGR placentas: median arterial length density was 14.82 mm/cm^3^ (IQR 13.13–18.10 mm/cm^3^) while median venous length density was 32.10 mm/cm^3^ (IQR 22.08–50.92 mm/cm^3^); p = 0.004 (Fig. 4b). In Fig. 5, we re-presented the length density data in a way to compare FGR to normal placentas. Arterial length density was significantly shorter at 14.82 mm/cm^3^ (IQR 13.13–18.10 mm/cm^3^) versus 27.29 mm/cm^3^ (IQR 19.65–42.44 mm/cm^3^); p = 0.009 (Fig. 5a), while venous length density was significantly longer at 32.10 mm/cm^3^ (IQR 22.08–50.92 mm/cm^3^) versus 13.39 mm/cm^3^ (IQR 12.18–22.94 mm/cm^3^); p = 0.03 (Fig. 5b) in the FGR group of placentas. The numerical density of vessel segments identified in each cast was not different. Similarly, there were no differences between the true branch density, no difference in density of vessel tortuosity (defined as total number of segments minus number of true branches), and no significant differences were demonstrable in arterial or venous diameter in either normal or FGR pregnancies above the measurement threshold of 100 µm. There was also no significant difference in the median diameter of the umbilical vessels in the casts (Supplementary Table 1).

**Figure 6:**
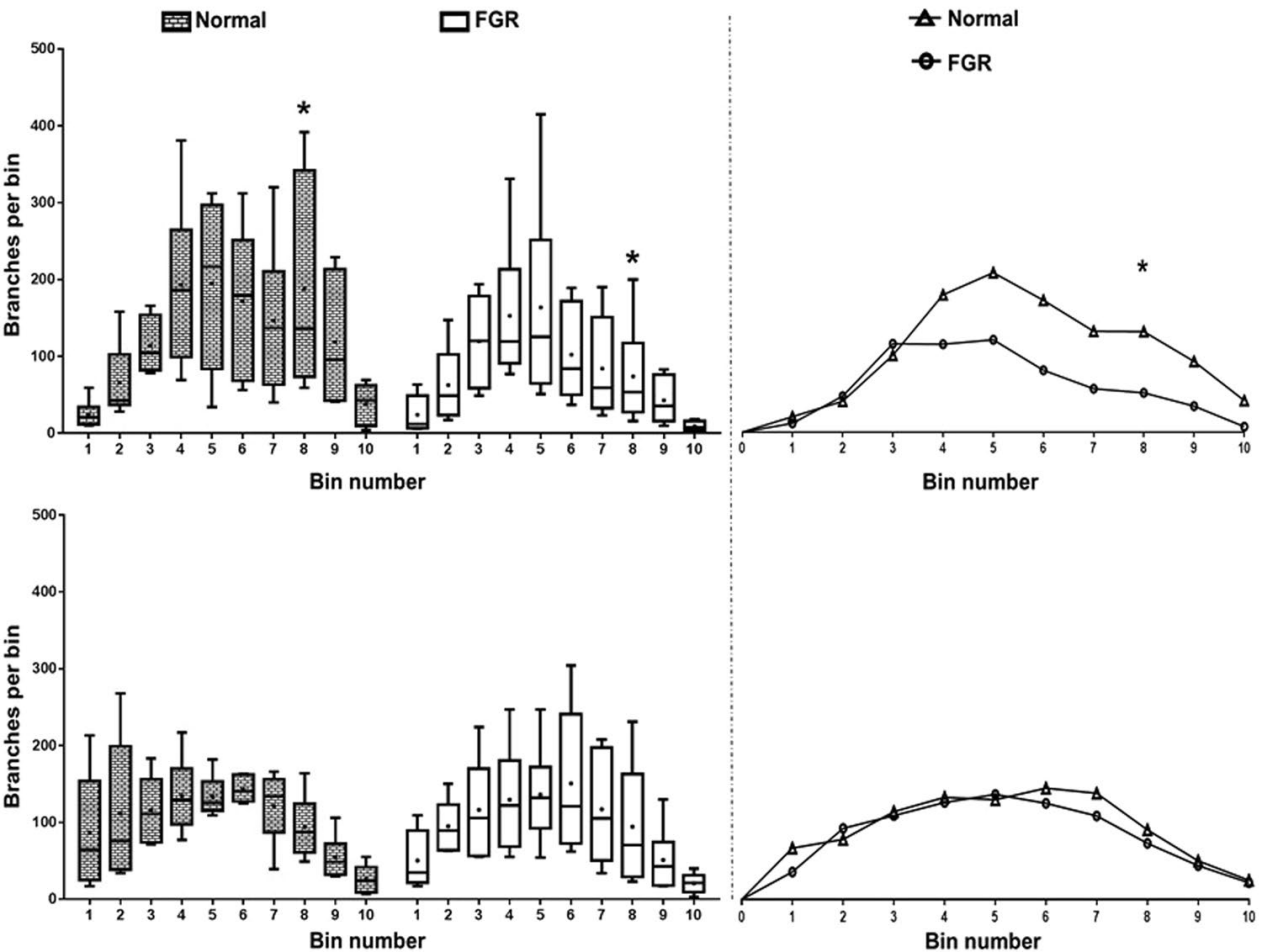
The number of branches in each of the ten bins in the tree maps. Graphs represent arterial (top left) and venous (bottom left) tree maps. Line and dots within the boxes represent the median and mean respectively. There are significantly fewer branches in the FGR arterial trees around the level of the 8^th^ bin of branches compared to normal while there is no difference in the number of branches in the venous trees. Curves on the top and bottom right represent the relationship between the median number of branches in the arterial and venous trees respectively. There is a trend suggesting fewer number of branches in the 4^th^–10^th^ bins of branches in the FGR arterial trees (top right). This trend is absent in the venous trees (bottom right). Data presented as boxplots showing the median, range and interquartile range. n = 12 normal (6 arterial, 6 venous) and 12 FGR (6 arterial, 6 venous) casts. Two-way RM Anova with Sidak’s multiple comparison test, alpha value = 0.05, *marks two-tailed *p* value = 0.002.

In an attempt to identify the exact region where vascular lengths differ along the course of the vessels as they ramified in the FGR and normal placentas, we grouped the branch levels in the Analyze tree maps into deciles of branching (Fig. 3b) and compared the number of branches at each decile level in the FGR trees to those in the normal trees. The number of branches in the arterial trees differed significantly at the eighth decile group (Fig. 6), in addition to a trend that suggests fewer branches from the fourth to the tenth decile group of branches in the FGR arterial trees. These differences were not seen when the venous trees were compared.

## Discussion

Intact human fetoplacental vascular topology has previously been poorly studied. Our creation of a three-dimensional digital representation of most of the fetoplacental vascular tree in intact state addresses this issue. We have previously demonstrated that corrosion casting on its own can provide vessel structure information on either the fetal arterial or venous trees, but this method struggles with the examination of smaller vessels^7^. Computer aided analysis now allows the use of advanced imaging techniques for resolving and quantifying the fine architecture of small calibre vessels^21–23^. Micro-CT has been used in the study of anatomical beds such as the kidney glomeruli^10, 11^, coronary branches in the heart^12^ and mouse placental vasculature^14, 24^, as well as vessels in selected small regions of human placenta^21, 25^.

In this study, we have demonstrated the potential of a combination of corrosion casting and microcomputed tomography (micro-CT) imaging to achieve a fuller account of human placental vascular morphology. We achieved resolution of vessels down to ~80 µm (diameter) when a whole cast measuring ~17 cm in its widest diameter was scanned. In the placenta, vessels at this diameter are at the terminal arteriole or post-capillary venule level. Below this level are the capillaries, known to measure up to 50 µm when visualised by microscopy of semithin sections^26^. Our current method, therefore, excludes the capillaries. Histology can provide detailed information on the capillaries and surrounding tissues, though, it is laborious and reliant on sampling of representative areas from what can often prove to be a heterogeneous vascular bed, especially in the context of placental pathology^27^. Micro-CT of the whole placenta demonstrates that above the level of terminal capillaries there are more small-diameter arteries than veins (Fig. 2); this allowed a more accurate comparison of vessel morphometry between normal and abnormal pregnancies. For the first time we have demonstrated longer venous and shorter arterial vasculature in FGR placentas. We have previously demonstrated the presence of fewer chorionic plate arteries^7^ and sparse capillaries^7, 28^ in FGR placentas. Our findings are in agreement with other reports of vessel length discrepancies observed between villi specimens from normal and FGR placentas compared by electron microscopic examination of vessel casts^29^ as well as by stereological analyses of histologic specimens^30, 31^. Previous comparisons, however, majorly involved the villi vessels while our approach has permitted comparison of a fuller vasculature as well as comparison of arterial and venous vessels separately.

Although delivery was significantly earlier in the FGR cases, seven of these cases attained term before delivery while five were delivered 10–14 days before term (between 252–256 days). It is unlikely for the placental vasculature to change significantly within this short period, therefore, we do not expect the difference in gestational age would impact on our findings. Our Analyze generated branching trees identified variation between normal and FGR placentas in distal, but not terminal portions of the fetal arterial tree (at the 8^th^ decile of branching level). However, Analyze cannot identify the spatial distribution of vessel branches in placental casts and consequently; we were unable to define exact vessel location. This means that vessel diameters at different segment distributions cannot be identified and although the differences noted are clearly near the terminal end of the villous tree (Fig. 6), we cannot specify at which exact diameters this occurs. Previous studies examining human fetal placental venous structure are sparse, though some reports have linked alterations in umbilical vein structure to FGR^32, 33^. The consequences of these previously unreported altered vessel path lengths on blood flow to the fetus are unknown. Given that placental veins convey oxygenated blood and nutrients to the fetus, it may be that longer venous return path in FGR pregnancies reduces the efficiency of materno-fetal nutrient transfer. Indeed, predictions from mathematical models of villous geometry are that altered vessel lengths negatively impacts on materno-fetal oxygen exchange and transfer^34^ and, in *ex vivo* perfusion studies, FGR placental vessels demonstrated high resistance to blood flow^35^. Alternatively, the increased venous length may be an angiogenic response to lower oxygen levels in the FGR placenta.

We were able to determine the tortuosity/loopiness of the vessels in the networks. Capillaries in FGR placentas have previously been demonstrated to have poorly coiled, elongated loops^29^, with reduced or similar tortuosity of individual villous branches compared to normal^36^. Our data adds that loopiness is found at all levels of the placental vascular tree (not just the villi), and overall, there are no differences in loopiness in normal and FGR placental vessels. This finding, together with our finding of similar vessel branch density in the normal and FGR vessel casts, suggests that the discrepancies we observed in vessel length were not due to variations in number of branches or loopiness of the vessels in the placentas.

The strength of our study is the demonstration of whole organ vascular differences however there are some limitations of the current method employed. Firstly, although vessels ~80 µm could be visualised, smaller arterioles, capillaries and post-capillary venules could not be seen. Secondly, using our current technique arterial and venous networks cannot be examined in the same placenta. This partly relates to the casting material used and further experiments using radio-opaque microfil may address this. Thirdly, although we were able to adapt the Avizo and Analyze software for analyses of placental vasculature, both software are not primarily designed for this purpose and so were not completely comfortable with the dataset. This is a major challenge in 3D placental research as there is currently no software designed specifically for placental vessel morphometry. The branch trees generated by the Analyze programme are ineffective for anatomical localisation of vessels, a parameter that could help pin-point the exact vessels involved in the differences identified and heterogeneity of vascular ramification in relation to the position within the organ. We also were not able to examine branching angle distributions which, in terminal branches of villous trees, have been reported to relate inversely to feto-placental weight ratio^37^ and have been found to differ in normal and FGR terminal villi^36^. In our study, normal umbilical artery diameter was 21% less and normal umbilical vein diameter was 36% less than diameters measured at the same gestational age *in vivo* by ultrasound^38^. Although differences in measurement site (we measured umbilical vessel diameters near the placental insertion site, whilst reported *in vivo* measurements were taken near the fetal abdominal cord insertion site) may account for this in part, the reduced diameter *ex vivo* may reflect a contribution from elevated vascular tone, an anticipated limitation of *ex vivo* perfusion. Lastly, although the population studied was relatively tightly defined and statistical differences were observed, the cases were mostly late onset FGR and were likely to have included normal small fetuses and fetuses that are small due to non-vascular pathology. Further experiments in a more tightly defined early onset FGR group are planned.

Whole human organ vascular examination of a large organ such as the placenta is possible and when coupled with computer-aided analysis provides a method for the examination of multimodal vascular abnormalities in pregnancy disease and other pathologies where vascular abnormalities may occur. Abnormalities observed specifically in placentas from fetal growth restricted pregnancies confirm the abnormalities observed in vascular development occur at multiple levels of the vascular tree. This provides potential targeting information for the development of therapeutic agents.

## Electronic supplementary material

Supplementary Table 1
