# Supplementary material for: Whole organ vascular casting and microCT examination of the human placental vascular tree reveals novel alterations associated with pregnancy disease
Source: Sci Rep. 2017 Jun 23;7:4144. doi: 10.1038/s41598-017-04379-0 (PMC5482861; doi:10.1038/s41598-017-04379-0)
Supplement: Supplementary file 1 — Supplementary Table 1 [file 41598_2017_4379_MOESM1_ESM.pdf]

Whole organ vascular casting and microCT examination of the human placental vascular tree reveals novel alterations associated with pregnancy disease

Toluwalope O Junaid[a*], Robert S Bradley[b], Rohan M Lewis[c], John D Aplin[a] and Edward D Johnstone[a]

[a]Maternal and Fetal Health Research Centre, Institute of Human Development, Faculty of Medical and Human Sciences, University of Manchester, Manchester, United Kingdom.

[b]Henry Moseley X-ray Imaging Facility, School of Materials, University of Manchester, United Kingdom.

[c]Faculty of Medicine, University of Southampton, Southampton, United Kingdom.

Supplementary Table 1: Number of branches, length, tortuosity and diameter of vessels in the normal and FGR vascular casts.

	Normal Arterial (n = 6)	FGR Arterial (n = 6)	***p***	Normal Venous (n = 6)	FGR Venous (n = 6)	***p***
Total vessel length per placenta (mm)[†]	17823(11287-24614)*	5947 (5294 – 7247)*	**0.002**	8188 (7026 – 10446)	9395 (7334 – 13853)	0.818
Length density (mm/cm^3)	27.29 (19.65 – 42.44)*	14.82 (13.13 – 18.10)*	**0.009**	13.39 (12.18 – 22.94)*	32.10 (22.08 – 50.92)*	**0.03**
Number of segments[†]	6714 (5563 – 16280)*	3379 (2389 – 5035)*	**0.009**	5523 (4225 – 7664)	5142 (4321 – 5783)	0.39
Numerical density (per cm^3)	10.86 (9.09 – 27.72)	9.23 (6.83 – 11.20)	0.18	10.76 (6.32 – 15.15)	13.01 (11.30 – 25.90)	0.39
Total number of branches[Ω]	2493 (1324 – 3550)	1458 (815 – 2657)	0.24	2009 (1621 – 2475)	1883 (1374 – 2431)	0.59
Branch density (per cm^3)	4.21 (2.31 - 5.68)	3.19 (2.32 - 6.53)	0.94	4.12 (2.42 - 5.64)	5.81 (4.69 - 7.91)	0.13
Number of branch points[Ω]	1241 (659 – 1766)	727 (406 – 1323)	0.24	1001 (809 – 1233)	938 (685 – 1212)	0.59
Branch density (per cm^3)	2.09 (1.15 – 2.83)	1.59 (1.15 – 3.25)	0.94	2.06 (1.21 – 2.81)	2.89 (2.34 – 3.94)	0.13

Number of terminal branch points$^{\Omega}$	1252 (666 – 1784)	731 (409 – 1334)	0.24	1008 (812 – 1243)	945 (689 – 1219)	0.59
Branch density (per cm^3)	2.11(1.16 - 2.86)	1.60 (1.17 - 3.28)	0.94	2.07 (1.21 - 2.83)	2.91 (2.36 - 3.97)	0.13
Number of branch levels$^{\Omega}$	71 (41 – 93)	46 (38 – 74)	0.48	129 (84 – 194)	76 (67 – 152)	0.39
Branch density (per cm^3)	0.12 (0.07 - 0.15)	0.13 (0.10 - 0.17)	0.59	0.28 (0.12 - 0.42)	0.34 (0.15 - 0.55)	0.48
Tortuosity	5631 (4074 – 14903)*	2421 (1767 – 4392)*	**0.03**	5562 (3774 – 6741)	5593 (4322 – 14458)	0.82
Numerical density (per cm^3)	9.66 (6.19 – 25.39)	7.52 (4.34 – 10.13)	0.33	8.92 (6.66 – 13.89)	14.61 (10.64 – 32.02)	0.13
Diameter† (µm)	2374 (2060 – 2785)	2128 (1824 – 3263)	0.59	3466 (2648 – 5255)	2123 (1014 – 3307)	0.13
Diameter density (µm/cm^3)	4.00 (3.12 – 4.73)	5.94 (4.11 – 8.29)	0.13	5.68 (3.89 – 13.73)	5.97 (3.82 – 7.99)	0.82
Umbilical vessel diameter† (µm)	3810 (3105 – 4630)	3232 (2610 – 4368)	0.48	5746 (4999 – 6862)	5225 (2759 – 5924)	0.24

Data shown are median and interquartile range (IQR) in parenthesis. For diameter, data shown are median and IQR of the mean diameter of all vessel segments in the casts. Umbilical vessel diameter refers to the diameter of the largest vessel segment in the casts. † and Ω denote data generated on Avizo and Analyze software respectively. Data compared by Mann-Whitney test, * denotes $p<0.05$.
